# Charge-Based Inhibitors of Amylin Fibrillization and Toxicity

**DOI:** 10.1155/2015/946037

**Published:** 2015-10-20

**Authors:** Sharadrao M. Patil, Andrei T. Alexandrescu

**Affiliations:** Department of Molecular and Cell Biology, University of Connecticut, 91 N. Eagleville Road, Storrs, CT 06269-3125, USA

## Abstract

To test the hypothesis that electrostatic repulsion is an important force opposing amyloid fibril assembly, we designed peptides that substitute strings of positively or negatively charged residues into the sequence of the amyloidogenic hormone amylin, which contributes to type 2 diabetes pathology. Arg-1 and Arg-2 substitute four positively charged arginines for segments that in structural models of amylin fibrils form the end of strand *β*1 and the beginning of strand *β*2, respectively. Mem-T substitutes negatively charged aspartates for the peptide segment with the largest avidity for membranes. All three charge-loaded peptides fibrillize poorly on their own and inhibit fibril elongation of WT-amylin at physiological ionic strength. The inhibition of WT-amylin fibril elongation rates is salt-dependent indicating that the analogs act through electrostatic interactions. Arg-1 protects against WT-amylin cytotoxicity towards a MIN6 mouse model of pancreatic *β*-cells, and Arg-2 protects at higher concentrations, whereas Mem-T has no effect. The most effective variant, Arg-1, inhibits WT-amylin fibril elongation rates with an IC_50_ of ~1 *µ*M and cytotoxicity with an IC_50_ of ~50 *µ*M, comparable to other types of fibrillization inhibitors reported in the literature. Taken together, these results suggest that electrostatic interactions can be exploited to develop new types of inhibitors of amyloid fibrillization and toxicity.

## 1. Introduction 

Consideration of amyloid structures suggests that like-charges, replicated along the fibril axis by the intermolecular *β*-sheet pairing of monomers, should energetically disfavor self-association due to electrostatic repulsion [1, 2]. Conversely, compensation of charges displayed on fibril surfaces may be important in the interactions of amyloids with polyanions such as heparan sulfate proteoglycans and membrane lipid bilayers [1, 3, 4]. Replacements of single charged residues can have large effects on fibrillization kinetics attesting to the important roles of charges in fibril assembly [2, 5–7].

In type 2 diabetes, the positively charged 37-residue hormone amylin misfolds into cationic fibrils which have been implicated in the destruction of the pancreatic *β*-cells that make insulin and amylin, thus contributing to pathology [8]. Amylin is a particularly favorable system for investigating the roles of charges in fibrillization, since the core of the intermolecular *β*-sheet fibril structure has only one pH-titratable group, His18 [9]. The histidine acts as an electrostatic switch, inhibiting fibrillization in its charged state at acidic pH and favoring fibrillization in its uncharged state at neutral pH [9–11]. The charged state of His18 affects fibril morphology as determined by TEM [9–11]. Substitution of a positively charged arginine at position 18 lowers cytotoxicity to MIN6 models of pancreatic *β*-cells compared to WT-amylin, which has an uncharged histidine at a physiological pH of 7.4 [9, 12]. Similarly, amylin with the S20K mutation fibrillizes much more slowly, in part due to the introduction of a positive charge in a segment of the peptide that participates in the hairpin turn of the fibril structure [7]. Moreover, the S20K mutant peptide inhibits fibrillization of WT-amylin when added in* trans* [7].

**Figure 1:**
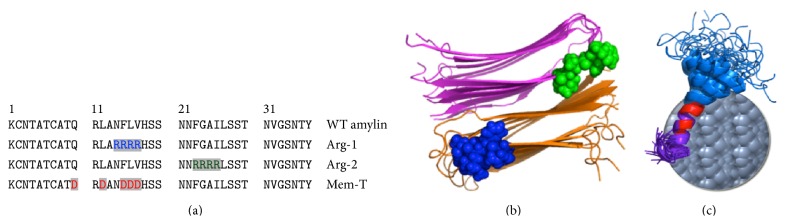
Design of fibrillization inhibitors. (a) Sequences of WT amylin and the three peptide analogs that incorporate strings of positive or negative charges in the amylin sequence. (b) Model of the stacked *β*-hairpin structure of amylin fibrils based on ssNMR [13]. Positively charged arginine residues are shown as spheres that are positioned at the end of strand *β*1 in Arg-1 (blue) or at the start of strand *β*2 in Arg-2 (green). (c) Solution NMR model of micelle-bound amylin, in which the N-terminal residues 5–17 are embedded in the hydrophobic environment of the micelle [16]. The Mem-T peptide substitutes hydrophobic residues in this region for five aspartates (red), in order to interfere with membrane binding through electrostatic repulsion between negatively charged residues on the peptide and lipid head-groups.

These observations suggest that the introduction of single charged amino acids in the portions of the amylin sequence that form the fibril core can markedly inhibit fibrillization. We therefore thought to exploit these properties by designing peptide variants that incorporate a string of residues with like-charges in the amylin sequence, as shown in Figure 1. The first peptide, arginine-variant 1 (Arg-1), substitutes four arginines for WT-amylin residues Asn14-Val17 (Figure 1(a)). These residues form part of strand *β*1 (blue spheres in Figure 1(b)) in the model of the amylin fibril structure determined by ssNMR [13]. The protofilament building block of the amylin fibril structure [13] has two C_2_-symmetry related stacks of intermolecular *β*-sheets (shown in orange and purple in Figure 1(b)). The positively charged residues introduced in the Arg-1 variant would be positioned on the surface of the protofilament. In our design, we envisioned that Arg-1 would act as a fibril extension inhibitor. The highly amyloidogenic segment between residues Ser20-Ser29 [8, 14] is retained in the sequence and would allow the peptide to attach to growing fibrils of WT-amylin, whereas the four arginines in Arg-1 would disfavor addition of monomers through electrostatic repulsion with the positively charged residues such as Arg11 and His18 in the WT-sequence. It is important to note that since the C-terminus of amylin is naturally amidated, there are no negatively charged residues in the sequence of WT-amylin. In a second analog, arginine-variant 2 (Arg-2), four arginine residues are substituted for residues Phe23-Ile26 in the center of the amyloidogenic segment. In the fibril structure [13] this corresponds to the end of the *β*-hairpin and the start of strand *β*2 (green spheres in Figure 1(b)). The design objectives for Arg-2 were the same as for the Arg-1 but the string of four arginine residues is positioned in the interior of the protofilament structure as opposed to the surface (Figure 1(b)). A third peptide, the “Membrane Trojan” analog (Mem-T), was conceived as an inhibitor of the interactions of WT-amylin with cell membranes. The motivation for the design of Mem-T was that some studies have suggested that the critical species responsible for amylin toxicity may not be amyloid fibrils but soluble oligomers that form membrane-spanning pores, thus compromising intracellular ion homeostasis and cellular integrity [8, 15]. The Mem-T analog (Figure 1(c)) was based on our NMR structure of WT-amylin bound to membrane mimetic SDS micelles [16]. In the NMR structure, the Ala5-Val17 segment is positioned in the hydrophobic environment of the micelle based on paramagnetic probe studies [16]. This segment has the highest avidity for lipid membranes based on a number of studies [8, 17]. In the Mem-T analog, hydrophobic residues from the Ala5-Val17 segment are replaced by five negatively charged aspartates (Figure 1(a)). We envisioned that the Mem-T analog would be able to form mixed oligomers with WT-amylin, through the His18-Tyr37 segment which would be positioned on the surfaces of membranes but that membrane penetration of the mixed oligomers would be blocked through electrostatic repulsion between the negatively charged aspartates at the N-terminus of the Mem-T peptide and the negatively charged phosphate groups of the membrane lipid bilayer (Figure 1(c)).

In the present work, we examined the ability of the three amylin analogs Arg-1, Arg-2, and Mem-T to form fibrils using a kinetic assay that employs the amyloid-specific fluorescent dye thioflavin T (ThT) and by imaging the reaction products with transmission electron microscopy (TEM). We investigated the ability of peptide analogs to inhibit fibril formation when added in* trans* to WT-amylin and characterized the concentration dependence of inhibition. Because we expected the three designed peptides to exert their effects through electrostatic interactions, we also examined how salt concentration affects inhibition. Finally, we investigated the ability of the inhibitor peptides to suppress cytotoxicity in a MIN6 mouse model of *β*-pancreatic cells [18] challenged with WT-amylin.

## 2. Materials and Methods

### 2.1. Materials

Human WT-amylin was from Biopeptide (San Diego, CA). The Arg-1, Arg-2, and Mem-T peptides were custom-synthesized by NeoBioLab (Woburn, MA). All peptides were prepared by solid-phase synthesis and had an amidated C-terminus, which occurs as a natural posttranslational modification in human WT-amylin. The peptides were purified to >95%, supplied as lyophilized powders, and were taken up in 100% DMSO to form stock solutions that were stored in aliquots at −80°C before use. The peptide concentrations of the stock solutions were measured using the Micro BCA Protein Assay Kit (Thermo Scientific, Rockford, IL). Freshly thawed aliquots of the stocks were used to make solutions of the desired peptide concentration, which contained final DMSO (v/v) concentrations of 1% for cytotoxicity experiments and 2% for all other experiments. Ultrapure grade thioflavin T was from AnaSpec (Fremont, CA). The Alamar Blue dye to measure cell viability in cytotoxicity assays, FBS (fetal bovine serum), and DMEM (Dulbecco's Modified Eagle Medium) cell culture medium were from Invitrogen (Carlsbad, CA). All other chemicals were from Fisher (Pittsburgh, PA).

### 2.2. ThT Assays of Fibrillization Kinetics

The time course of fibrillization in solution was monitored using 100–200 *μ*L amylin samples, contained in white polystyrene clear bottom 96-well plates (Corning Inc., Corning, NY). Plates were covered with a clear polyester sealing tape (Fisher Scientific, Agawam, MA) to prevent evaporation. Stock solutions of 1.1 mM WT-amylin and inhibitor peptides were prepared in 100% DMSO, which dissolves and disaggregates amylin fibrils [20], and were stored at −80°C when not in use. Starting from the 1.1 mM stock solutions in 100% DMSO, samples for fibrillization reactions were prepared to contain 20 *μ*M amylin and 10 *μ*M ultrapure ThT, in 20 mM sodium phosphate buffer, pH 7.4, and a final DMSO concentration of 2% (v/v). For experiments using larger 400 *μ*M concentrations of Arg-2 and Mem-T, the solutions were prepared by diluting 100% DMSO stock solutions of 20 mM peptide to a final DMSO concentration of 2% (v/v). Amylin was the last component added to the samples for the kinetic reactions, in order to reduce the dead time for the experiments. The plates were incubated at 25°C without agitation. Fluorescence intensity was recorded at 2 min intervals with excitation at 440 nm and emission at 490 nm on a Fluoroskan Ascent 2.5 fluorescence plate reader. Fibrillization reactions for the peptides alone were performed in triplicate and for analog-peptide inhibition of WT-amylin in duplicate, to estimate experimental uncertainties in kinetic parameters.

### 2.3. Transmission Electron Microscopy

Samples containing 80 *μ*M concentrations of WT-amylin and the three analogs were incubated without agitation at a temperature of 37°C in 20 mM phosphate buffer (pH 7.4). For the inhibition reactions, samples contained WT-amylin at an 80 *μ*M concentration, together with 160 *μ*M of Arg-1, Arg-2, or Mem-T analogs. Aliquots from the reactions were removed after 2 days for TEM imaging. The aliquots were blotted onto carbon-coated 400-mesh Maxtaform copper grids (Ted Pella Inc. Redding, CA) for 1–3 min, followed by negative staining with 1% uranyl acetate. TEM images were recorded on an FEI Tecnai G^2^ Spirit BioTwin transmission electron microscope equipped with an AMT XR-40 camera.

### 2.4. Cytotoxicity Assays

Amylin samples were prepared by dissolving lyophilized peptides in 100% DMSO to 8 and 12 mM concentrations for WT-amylin and the analogs, respectively, as determined with the micro-BCA protein assay kit. The stock solutions were diluted with FBS-free DMEM and sonicated continuously for 5 min at 75% amplitude before use. FBS was subsequently added to a concentration of 15% (v/v), giving final amylin concentrations of 40, 80, and 160 *μ*M. The final DMSO concentration for all cytotoxicity experiments was 1% (v/v).

Cytotoxicity was measured using the mouse insulinoma 6 (MIN6) cell line model of *β*-pancreatic cells [18], which were a gift from Dr. Anil Rustgi (University of Pennsylvania). Cells were seeded at a density of 20,000 per 100 *μ*L in black clear-bottom 96-well plates. The cells were grown in DMEM with 15% FBS, 25 mM glucose, 2 mM L-glutamine, 500 mM sodium pyruvate, 55 *μ*M *β*-mercaptoethanol, 1000 units/mL penicillin, and 100 *μ*g/mL streptomycin, for 20 h at 37°C in a humidified incubator with 5% CO_2_. The culture medium was then removed and replaced with fresh medium containing WT-amylin and/or inhibitor peptides. The cells were incubated for another 24 h followed by the addition of 10% (v/v) of the redox indicator dye Alamar Blue at the concentration supplied by the manufacturer (Invitrogen). Fluorescence, due to the reduction of Alamar Blue by viable cells, was measured after 6 h, using excitation and emission wavelengths of 544 and 590 nm, respectively. Cell viability was calculated from the ratio of Alamar Blue fluorescence in treated to untreated cells. Uncertainties were calculated as the SEMs of triplicate measurements.

## 3. Results

### 3.1. Incorporation of Charged Residues Inhibits Fibrillization

**Figure 2:**
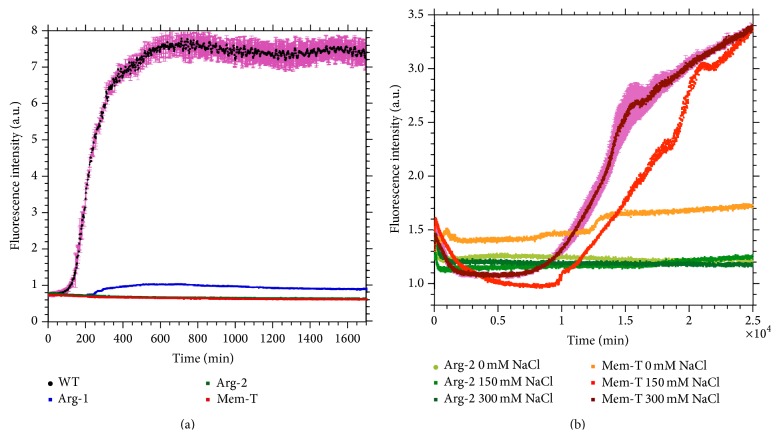
Fibrillization kinetics of WT and charged amylin variants. (a) Reaction profiles for 20 *μ*M peptide concentrations in the presence of 150 mM NaCl. (b) For the two variants Arg-2 and Mem-T that failed to fibrillize at 20 *μ*M peptide concentrations, aggregation was also studied at a larger 400 *μ*M peptide concentrations and the indicated salt concentrations. Representative error bars, calculated as the SEM from triplicate measurements, are shown for WT-amylin in (a) and for Mem-T at 300 mM NaCl in (b).

The three amylin analogs Arg-1, Arg-2, and Mem-T considered in this work substitute strings of like-charged amino acids for segments of the amylin sequence. We first compared fibrillization of the analogs and WT-amylin (Figure 2). At a physiological salt concentration of 150 mM NaCl and peptide concentration of 20 *μ*M, we could only detect fibrillization for WT-amylin and Arg-1. The change of ThT fluorescence between the start and steady-state plateau of the reactions is 30-fold larger for WT-Amylin compared to the Arg-1 peptide (Figure 2(a)). The lag time for the Arg-1 analog (210 min) is increased about 2-fold compared to WT-amylin (120 min) while the elongation rate for Arg-1 (0.0068 min^−1^) is reduced about 30% compared to WT-amylin (0.010 min^−1^). With a 20-fold higher peptide concentration of 400 *μ*M, we observed weak fibrillization of Mem-T (Figure 2(b) orange to brown) but Arg-2 still failed to fibrillize (Figure 2(b) light to dark green). The fibrillization of Mem-T at the larger 400 *μ*M peptide concentration was salt dependent. In the absence of salt only a very weak signal for fibrils was detected (orange in Figure 2(b)). Fibrillization was stimulated at physiological salt concentrations and above (red and brown in Figure 2(b)), as expected for a mechanism in which charge-repulsion for the Mem-T analog is abated when the charges become screened by salt. At 150 mM NaCl, the fibrillization of 400 *μ*M Mem-T (lag time of 10,000 min, elongation rate of 6.1 ± 1.4 × 10^−6^ min^−1^) was still much weaker than for WT-amylin at a 20 *μ*M peptide concentration (lag time 120 min, elongation rate of 0.0100 ± 0.0001 min^−1^). The Arg-2 peptide did not form fibrils under any of the conditions tested (Figure 2(b)).

**Figure 3:**
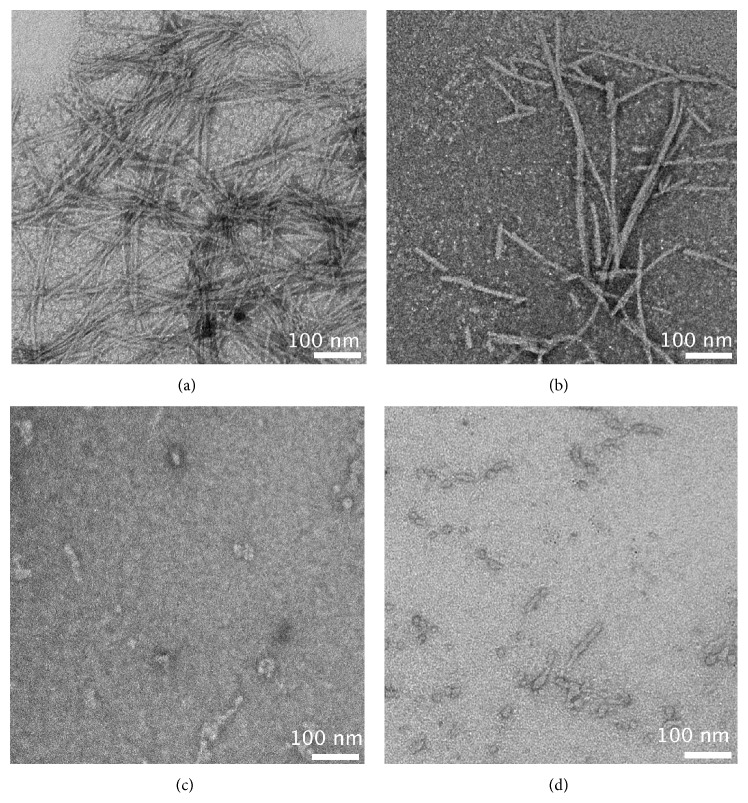
TEM images of aggregates formed by WT-amylin and charged variants. (a) WT amylin, (b) Arg-1, (c) Arg-2, and (d) Mem-T, after incubation for 2 days in 20 mM phosphate buffer, pH 7.4, at a temperature of 37°C. All peptide concentrations were 80 *μ*M.

EM images of the aggregates present after 2 days were consistent with the kinetics data (Figure 3). WT-amylin formed large amounts of fibrils (Figure 3(a)). By contrast Arg-1 formed much fewer fibrils; the section of the grid shown in Figure 3(b) has a relatively high number, to aid visualization. The image in Figure 3(b) clearly shows that Arg-1 formed a larger proportion of short fibrils than WT-amylin. For Arg-2 (Figure 3(c)) and Mem-T (Figure 3(d)) we only detected amorphous aggregates with nonfibrillar morphologies.

Taken together, these observations indicate that the introduction of charged residues in amylin analogs strongly interferes with their ability to form fibrils, as manifested by increased lag times and reduced fibrillization rates in kinetic assays (Figure 2) of the charge-loaded analogs. TEM imaging shows that compared to WT-amylin, Arg-1 forms fewer fibrils with shorter lengths, while Arg-2 and Mem-T form few if any fibrils (Figure 3).

### 3.2. Charge-Loaded Peptide Analogs Inhibit Fibrillization of WT-Amylin

**Figure 4:**
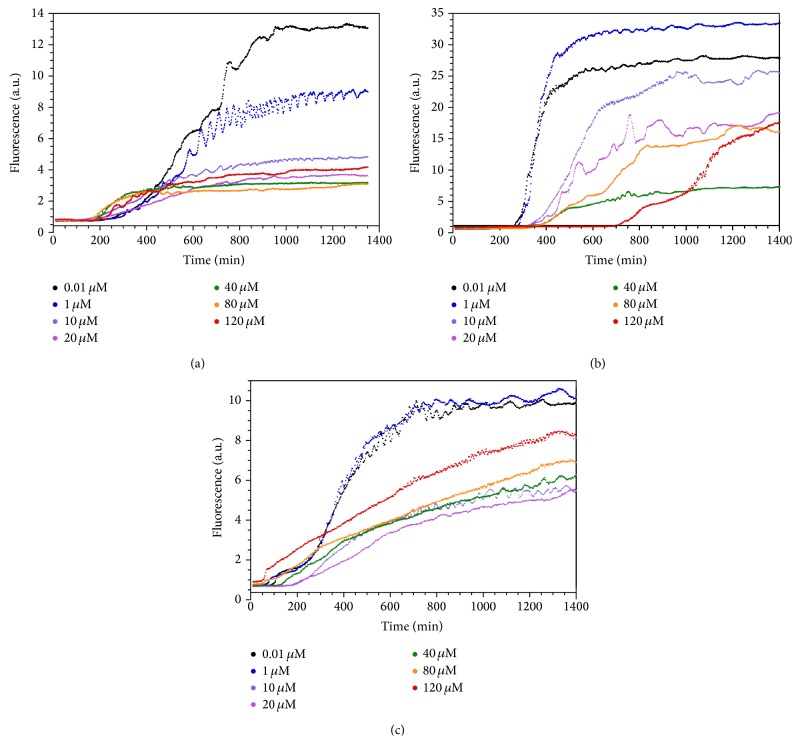
Representative reaction profiles showing the effects of peptide inhibitors at the indicated concentrations on the fibrillization of 20 *μ*M WT-amylin: (a) Arg-1, (b) Arg-2, and (c) Mem-T. All reactions were done in the presence of 150 mM NaCl. A single-step fibril-formation process was assumed for the analysis of all the kinetic reactions. Although some of the reactions appear to show more complicated fluctuations in the data, these are likely experimental noise (the presence of particulate fibrils leads to nonideality in fluorescence measurements) as they are not observed in replicate measurements.

We next examined whether the charge-loaded analog peptides affected the fibrillization of WT-amylin when added in* trans*.  Figure 4 shows representative kinetic traces from experiments in which the concentration of WT-amylin was fixed at 20 *μ*M while the concentration of the charge-loaded analogs was varied. In spite of their poor ability to fibrillize on their own, each of the three analogs affects the kinetics of WT-amylin fibrillization indicating that the analogs interact with the WT peptide. The most readily apparent effect is that fibrillization rates are reduced with increasing concentration of the analogs, manifested by a reduction in the slopes of the growth part of the reactions compared to WT-amylin alone. With Arg-1 there is also a reduction in the steady-state fluorescence plateaus with increasing concentration of the inhibitor (Figure 4(a)). Fibrillization lag times are increased with increasing concentrations of the Arg-2 peptide (Figure 4(b)) but decrease at high concentrations of the Mem-T analog (Figure 4(c)).

**Figure 5:**
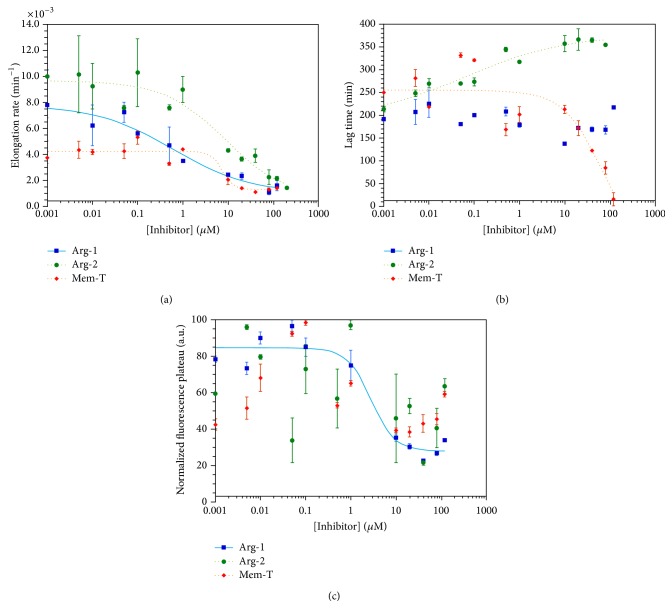
Effects of peptides added* in trans* on parameters describing the fibrillization kinetics of 20 *μ*M WT amylin: (a) elongation rates, (b) lag times, and (c) fluorescence plateaus. Inhibition data from Arg-1, Arg-2, and Mem-T are shown in blue, green, and red, respectively. All experiments were done in duplicate in the presence of 150 mM NaCl. The data points are average values for the kinetic parameters, and the uncertainty bars are SEM values calculated from duplicate reactions. Curves (where the data could be fitted) represent four-parameter nonlinear least squares fits of the inhibition data to the IC_50_ equation [19].

Figure 5 shows the effects of analog peptide concentrations ranging between 0.001 and 120 *μ*M on the kinetic parameters for the fibrillization of 20 *μ*M WT-amylin. All three peptides reduce fibril elongation rates (Figure 5(a)). The Arg-1 and Arg-2 inhibitors cause an 8–10-fold reduction in the rates for WT-amylin fibrillization, as conceived in the design of the peptides as fibril-elongation inhibitors. Mem-T shows a smaller 4-fold reduction in elongation rates. Although Mem-T was conceived as an inhibitor of the interactions of WT-amylin with membranes, the substitution of a string of negatively charged aspartate residues in the N-terminal half of the amino acid sequence inhibits fibril elongation, probably by the same mechanism as the introduction of positively charged arginine residues in the Arg-1 and Arg-2 analogs. The fact that inhibition of fibril elongation is weaker with Mem-T than with the arginine analogs is likely a consequence of WT-amylin being an entirely cationic peptide with no negatively charged residues at neutral pH. Electrostatic repulsion should be stronger between the intrinsic positively charged sites in WT-amylin and the introduced positive charges in the two arginine inhibitors than with the negative charges in the Mem-T analog.

**Figure 6:**
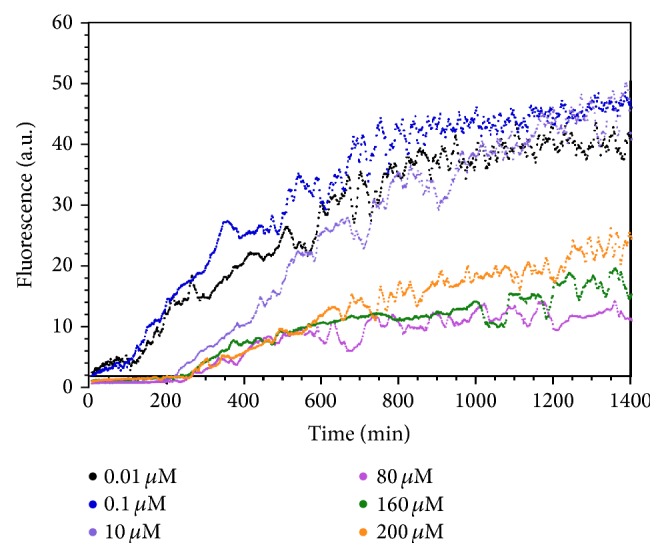
Representative kinetic traces for the fibrillization of WT-amylin at the larger peptide concentration used for the cytotoxicity assays (80 *μ*M amylin), and the indicated concentrations of the Arg-1 inhibitor.

**Figure 7:**
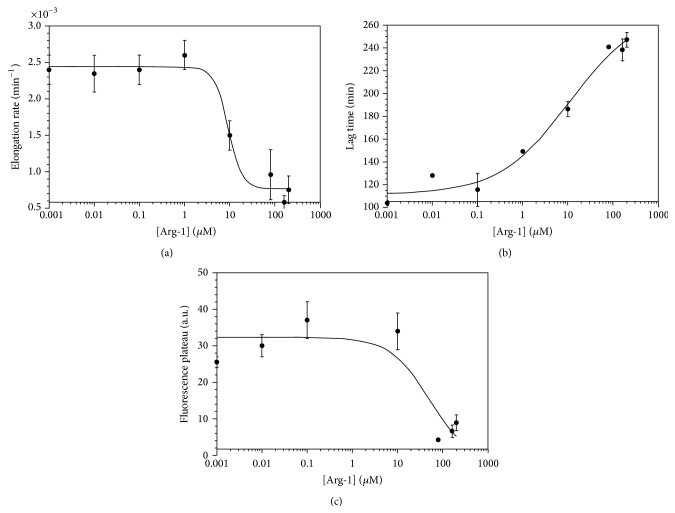
Dependence of kinetic parameters for the fibrillization of 80 *μ*M WT amylin (see Figure 6) on the concentration of Arg-1 inhibitor. Experiments were done in duplicate in the presence of 150 mM NaCl. Data points are average values for the kinetic parameters, uncertainty bars are SEM values from the duplicate reactions, and curves are four-parameter fits of the inhibition data to the IC_50_ equation. The IC_50_ values were 9.1 ± 5.2 *μ*M for the elongation rates (a), 11.0 ± 12.4 *μ*M for the lag times (b), and 50 ± 80 *μ*M for the steady-state fluorescence plateaus (c).

An IC_50_ analysis of the inhibition data was performed to obtain quantitative information (Figure 5). The Arg-1 concentration-dependence for the inhibition of WT-amylin fibril elongation rates gives an IC_50_ value of 0.6 ± 0.5 *μ*M. For Arg-2 and Mem-T, the IC_50_ values are ~10 *μ*M (Figure 5(a)). In addition to effecting elongation rates, Arg-2 increases the lag times for WT-amylin fibrillization with an IC_50_ of ~0.1 *μ*M. This indicates that Arg-2 inhibits the nucleation step of the reaction (Figure 5(b)). By contrast, Mem-T causes a reduction in lag times at high concentrations of the analog (>10 *μ*M) suggesting that it promotes the nucleation of WT-amylin. The half-maximal concentration for this effect was about 70 *μ*M. The reduction in lag times with Mem-T is reminiscent of what we previously observed with negatively charged heparin polysaccharides which enhance fibrillization of WT-amylin [4] and may occur because the negative charges in Mem-T complement the positive charges in the cationic amylin peptide, thereby facilitating fibril nucleation. With increasing Arg-1 concentration, there were no effects on the lag times within experimental uncertainty when the experiments were done with a 20 *μ*M concentration of WT-amylin (Figure 5(b)). When the reactions were done at a larger WT-amylin concentration of 80 *μ*M, the concentration used for cytotoxicity experiments (see below), we saw increases in lag times with increasing Arg-1 concentration as well as decreases in elongation rates and steady-state fluorescence plateaus (Figures 6 and 7). The effects of Arg-1 may be masked at the lower 20 *μ*M WT-amylin concentration, as fibrillization lag times increase with decreasing peptide concentration. Within experimental uncertainty, steady-state fluorescence plateaus were only observed to decrease with the Arg-1 analog, with an IC_50_ of 2.8 ± 1.7 *μ*M at 20 *μ*M WT-amylin (Figure 5(c)) or 49 ± 82 *μ*M (Figure 5(c)) at 80 *μ*M WT-amylin (Figure 7(c)).

**Figure 8:**
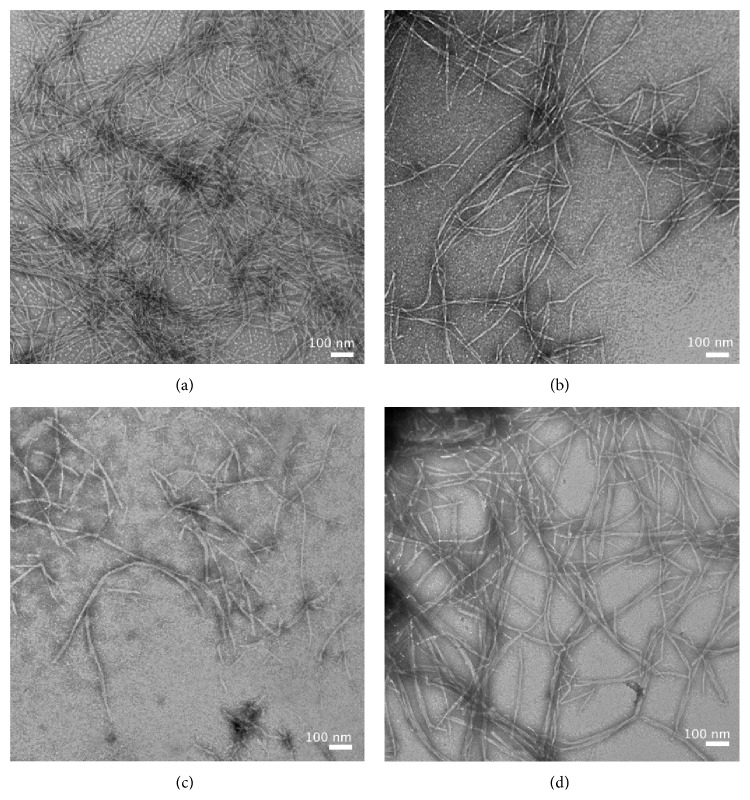
TEM images of fibrils formed from 80 *μ*M WT amylin in the presence or absence of inhibitor peptides. (a) WT amylin alone, (b) with 160 *μ*M Arg-1, (c) with 160 *μ*M Arg-2, and (d) with 160 *μ*M Mem-T. Aliquots were removed for TEM imaging 24 h after the fibrillization reactions were started. Reactions were carried out in 20 mM sodium phosphate (pH 7.4), 1% DMSO (v/v), at 37°C.

TEM imaging of WT-amylin fibrils formed in the presence of the charge-loaded analogs showed that the morphology of the fibrils is mostly conserved (Figure 8). In the presence of the least effective inhibitor Mem-T, the WT-amylin fibrils were indistinguishable from those formed with WT-amylin alone (Figures 8(a) and 8(d)). In the presence of the effective analogs Arg-1 (Figure 8(b)) and Arg-2 (Figure 8(c)), we observed somewhat fewer fibrils and a greater amount of short fibrils, compared to when WT-amylin was fibrillized alone. The increase in the amount of short fibrils is consistent with the greater potency of the Arg-1 and Arg-2 peptides as inhibitors of WT-amylin fibril elongation rates. Thus, while the Arg-1 and Arg-2 peptides do not stop fibrillization, they appear to inhibit fibril elongation as manifested by the smaller amounts of fibrils and the greater proportion of short fibrils in the presence of the inhibitors.

### 3.3. Salt Modulates the Inhibition of WT-Amylin Fibrillization by the Charge-Loaded Analogs

**Figure 9:**
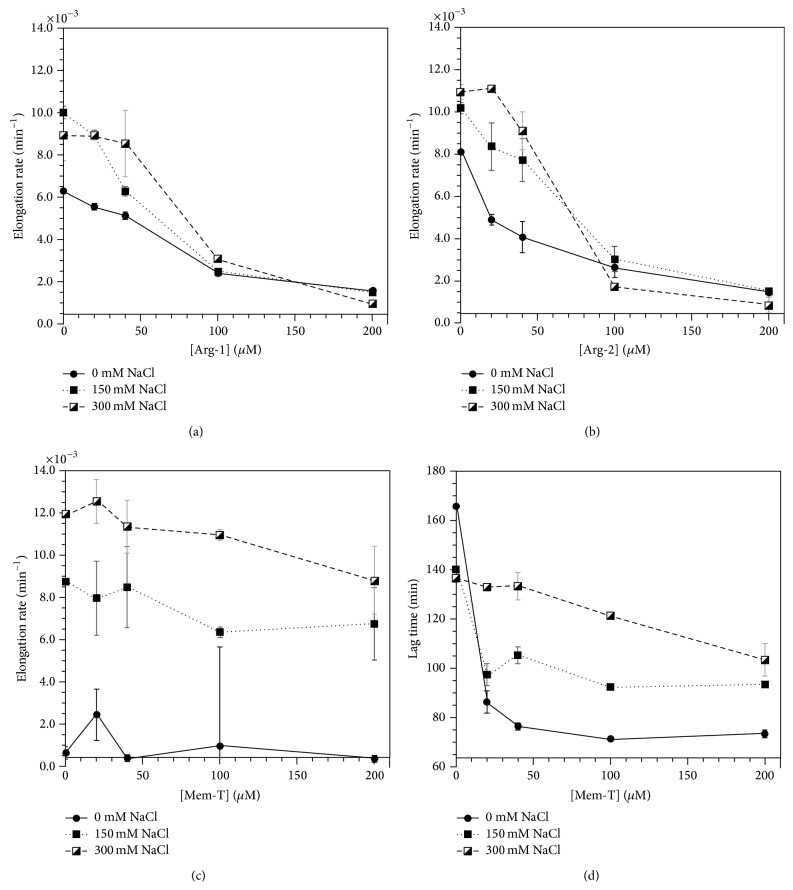
Salt-dependence on the effectiveness of charge-loaded analogs for the inhibition of 20 *μ*M WT-amylin fibrillization. Fibril elongation rates with Arg-1 (a), Arg-2 (b), Mem-T (c), and lag times with Mem-T (d).

Since we expected the charge-loaded amylin analogs to inhibit fibrillization through electrostatic repulsion we looked at the effects of salt, which should screen charges.  Figure 9(a) shows the effects of NaCl concentration on the most potent inhibitor Arg-1. Although we have too few data points to accurately determine IC_50_ values, the experiments clearly show that larger Arg-1 concentrations are required to decrease elongation rates as the salt concentration is increased. This is the expected result for an inhibition mechanism that involves electrostatic repulsion, as the charges become increasingly screened with increasing salt concentration. A very similar effect is seen with Arg-2 (Figure 9(b)) but with the least effective inhibitor Mem-T, salt effects on elongation rates are reduced close to the uncertainties of the measurements (Figure 9(c)). The shortening of fibrillization lag times with the Mem-T peptide, however, shows a strong salt concentration dependence indicating that the enhanced nucleation of WT-amylin fibrils in the presence of Mem-T occurs through electrostatic interactions (Figure 9(d)).

It is interesting to consider that the data in Figures 9(a) and 9(b) indicate that the reduction in elongation rates with Arg-1 and Arg-2 is more effective at physiological salt concentration or higher than in the absence of salt. This is because the fibrillization of WT-amylin, in the absence of any inhibitors, is enhanced with increasing ionic strength [21]. A 7-fold reduction in elongation rates is seen as the concentration of Arg-1 is increased between 0 and 200 *μ*M at 150 mM NaCl. In the absence of salt, there is only a 4-fold reduction over the same inhibitor concentration range (Figure 9(a)).

### 3.4. Arg-1 and Arg-2 Are Inhibitors of WT-Amylin Cytotoxicity towards *β*-Cells

**Figure 10:**
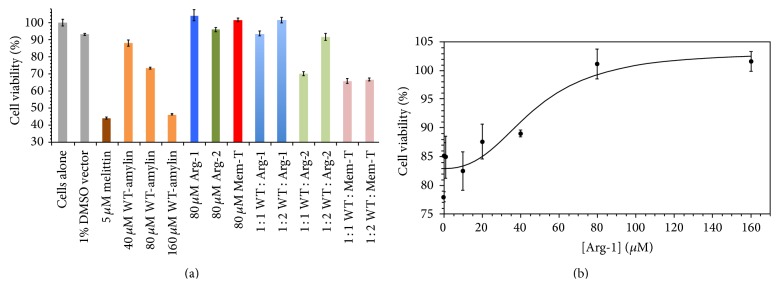
Effects of charge-based inhibitors on amylin cytotoxicity. (a) Cytotoxicity of WT-amylin and analogs towards a MIN6 mouse insulinoma cell model of *β*-pancreatic cells [18]. Gray, cells-only, defined as 100% cell viability and 1% DMSO vector used for all experiments except the first; brown, positive melittin control; orange, WT-amylin at different concentrations; blue, green, red-cells challenged with 80 *μ*M Arg-1, Arg-2, Mem-T respectively; light blue, green, red-cells challenged with 80 *μ*M WT-amylin and the indicated molar ratios of the respective inhibitors. (b) Viability of MIN6 cells challenged with 80 *μ*M WT-amylin as a function of Arg-1 concentration. The cell viability data were fit to an IC_50_ value of 47 ± 17 *μ*M Arg-1. All data points are averages from measurements performed in triplicate and the uncertainties are the associated SEMs.

We next looked at the effects of the charge-loaded amylin analogs on the cytotoxicity of WT-amylin towards a MIN6 model [18] of pancreatic *β*-cells (Figure 10). In control experiments, all three charge-loaded analog peptides show no toxicity towards the MIN6 cells (dark blue, green, and red in Figure 10(a)), giving viabilities comparable to untreated cells (gray in Figure 10(a)). We next did a concentration series challenging the MIN6 cells with 40, 80, and 160 *μ*M WT-amylin (orange in Figure 10(a)). As the concentration of WT-amylin is increased, cell viability drops to about 45% in the presence of 160 *μ*M WT-amylin, comparable to the value obtained with the potent toxin melittin from bee-venom, which was used as a positive control (brown in Figure 10(a)). We chose a 80 *μ*M concentration of WT-amylin for the inhibitor studies, as a compromise between detecting a sufficient signal in the assay (~25% cytotoxicity) and minimizing the WT-amylin peptide concentration, since this would require lower concentrations of the inhibitors to counteract the effects of WT-amylin. Of the inhibitors, Arg-1 analog protects against WT-amylin at a stoichiometric ratio of the two peptides: 80 *μ*M Arg-1 for MIN6 cells challenged with 80 *μ*M WT-amylin (light blue bars in Figure 10(a)). The Arg-2 analog is less effective than Arg-1 but protects against cytotoxicity at a 2 : 1 inhibitor : WT-amylin molar ratio (160 *μ*M Arg-2 : 80 *μ*M WT amylin, light green in Figure 10(a)). The Mem-T analog failed to protect against WT-amylin cytotoxicity (pink in Figure 10(a)).

For the most potent analog, Arg-1, we looked in detail at the inhibitor concentration dependence of cytotoxicity for MIN6 cells challenged with 80 *μ*M WT-amylin (Figure 10(b)). The inhibitor concentration data were fit with an IC_50_ value of 47 ± 17 *μ*M. This value is comparable to other WT-amylin cytotoxicity inhibitors reported in the literature, such as oligopyridylamide [22] and diarylated thiophene [23] *α*-helix mimetics (IC_50_ values of ~7 *μ*M and ~50 *μ*M estimated from the data in Figure 2(d) of [22] and Figure 3(b) of [23], resp.).

## 4. Discussion

The motivation for the studies described in this work was to see if electrostatic charge repulsion could be exploited to design new types of inhibitors of amylin fibrillization and cytotoxicity. Our work [9, 11] and that of others [10, 24, 25] have shown that charging of His18 in amylin at low pH can markedly inhibit fibrillization. The effects of charging His18 at low pH can be recapitulated in the H18R mutant of amylin at neutral pH, and this substitution results in an amylin peptide that is not cytotoxic to *β*-cells [9, 12]. Moreover, addition of a single charged lysine residue in the S20K mutation was reported to result in much slower fibrillization and to inhibit fibrillization of the WT-amylin peptide when the mutant peptide was added in* trans* [7]. Stimulated by these observations we designed three peptide analogs that substitute a string of 4-5 charged residues for neutral residues in the amylin sequence. The Arg-1 and Arg-2 analogs were designed as inhibitors of fibril elongation (Figure 1(b)). The Mem-T peptide (Figure 1(c)) was designed to interfere with membrane insertion of putative mixed Mem-T : WT-amylin oligomers. In this work we characterized the ability of the peptides to form fibrils by themselves, the concentration dependence of their inhibition of WT-amylin fibrillization, and their inhibition of WT-amylin cytotoxicity towards the MIN6 [18] mouse model of pancreatic *β*-cells.

In the cytotoxicity assays, Arg-1 was more potent than Arg-2 in protecting *β*-cells from WT-amylin, while the Mem-T analog offered no protection (Figure 10(a)). The origins of these differences are unclear but Arg-1 also serves as a more potent inhibitor of fibril elongation rates than Arg-2, with an IC_50_ of 0.60 ± 0.47 *μ*M for Arg-1, compared to 8.6 ± 8.2 *μ*M for Arg-2 (Figure 5(a)). The greater potency of Arg-1 compared to Arg-2 could be a structural effect. In the ssNMR model of amylin protofibrils [13] the four substituted arginines would be positioned at the surface of the structure in Arg-1, whereas they would be placed in the interior between the two C_2_-symmetry related stacks of *β*-sheets in Arg-2 (Figure 1(b)). Alternatively, the greater effectiveness of Arg-1 as an inhibitor may be related to its relatively better ability to form fibrils on its own, whereas Arg-2 did not form fibrils even at high concentrations of the peptide and salt. In other words, the capacity of Arg-1 to form fibrils although weakened compared to WT-amylin may make it better able to associate with the latter, thereby allowing it to better exert its inhibitory effects on fibril elongation.

The lack of protection against WT-amylin cytotoxicity with Mem-T could indicate that the design strategy of interfering with oligomer insertion into membranes did not work. Another possibility, since we do not know the optimum Mem-T : WT-amylin stoichiometry ratio for the putative mixed oligomers on which the design strategy was based, is that Mem-T could work at higher concentrations than the highest 2 : 1 Mem-T : WT-amylin ratio tested in this work. Like Arg-1 and Arg-2, Mem-T acts as an inhibitor of WT-amylin fibril elongation rates with an IC_50_ of 7.4 ± 6.6 *μ*M. The reduction in elongation rates with Mem-T is only about half of that for the arginine-peptides, and in contrast to the arginine peptides Mem-T decreases the lag times for WT-amylin fibrillization. The stimulation of the nucleation step for WT-amylin fibrillization, as manifested by the reduced lag times observed at high concentrations of Mem-T (Figure 5(b)), may be why this analog is ineffective as a cytotoxicity inhibitor. The enhanced fibril nucleation of WT-amylin at high concentrations of Mem-T is most likely due to the insertion of negative charges in this analog which could complement the positive charges in the WT peptide. An alternative way to design a peptide that could interfere with membrane insertion of mixed oligomers would be to disrupt the *α*-helix that interacts with the hydrophobic component of membranes (Figure 1(c)) by inserting prolines rather than negatively charged residues. This could have the desired effect of interfering with membrane insertion of mixed oligomers, while avoiding the stimulation of the nucleation of WT-amylin due to the negative charges in the Mem-T analog.

For the most effective analog Arg-1, we determined IC_50_ values of 1–10 *μ*M from the inhibitor concentration-dependence of the kinetic parameters for WT-amylin fibrillization (Figures 5 and 7). These values are comparable to those of other fibrillization inhibitors reported in the literature, for example, small molecules containing heterocyclic groups (IC_50_ = 1 *μ*M) [26] and *α*-helix peptidomimetics (IC_50_ = 8 *μ*M) [22, 23, 27]. We also looked at the Arg-1 concentration dependence of the inhibition of WT-amylin cytotoxicity and obtained an IC_50_ of 47 ± 17 *μ*M (Figure 10(b)). Although there is a dearth of similar studies for amylin inhibitors in the literature we were able to estimate a comparable IC_50_ of ~7 *μ*M from the data reported (Figure 2(d)) for the IS5 oligopyridylamide *α*-helix mimetic inhibitor of amylin cytotoxicity [22]. For the A*β*_1–42_ peptide involved in Alzheimer's disease, a number of different types of inhibitors give IC_50_ values in the range between 10 and 100 *μ*M in cell cytotoxicity assays [28–30]. A problem with the MIN6 cells used in this paper is that a relatively high concentration of WT-amylin is required to give a significant cytotoxicity signal, as shown by the concentration series represented by the orange bars in Figure 10(a). Under the conditions of this study (1% DMSO so as to not perturb cell membranes) 10%, 25%, and 50% cytotoxicity is achieved with WT-amylin concentrations of 40, 80, and 160 *μ*M WT-amylin, respectively. Because of the large amounts of WT-amylin necessary to detect a sufficient cytotoxicity signal, large concentrations of inhibitor peptides were needed to afford protection from WT-amylin. One possibility is to use another *β*-cell model, such as the INS-1 cell line but with this system as well, concentrations in the range between 5 and 50 *μ*M WT-amylin were required to give 50% cytotoxicity [22, 23]. A more sensitive cytotoxicity assay would allow the use of lower WT-amylin concentrations and possibly lower inhibitor concentrations.

It is currently uncertain which states of amyloidogenic proteins are harmful to cells. In the case of amylin, amyloid fibrils could exert their cytotoxic effects by perforating *β*-cell membranes or by disrupting the network of interactions with other cells in the islets (*α*, *ε*, *δ*, PP) that are necessary for the *β*-cells to function [8]. Many investigators have proposed that soluble oligomers rather than fibrils are responsible for the deleterious effects of amyloidogenic proteins. Annular oligomers could form membrane-spanning pores that would allow unregulated ion transport between the cell and its environment disrupting cellular homeostasis [8, 31, 32]. Because intermediates would be present at low concentrations and would be short-lived, oligomeric precursors to amyloids have proven difficult to isolate, and their properties are ill-defined [8, 33]. Oligomers also pose the difficulty that because they are transiently formed, they could interconvert to fibrils during cytotoxicity measurements making a definite assignment of their role in pathology equivocal [8, 34]. In yet another proposed mechanism, it is not the oligomers or fibrils themselves, but the process of fibril growth that could be responsible for cytotoxicity, by inducing membrane damage [35]. For the three analogs described in the paper, we do see a positive correlation between inhibition of fibril elongation rates and protection against WT-amylin cytotoxicity to *β*-cells. Arg-1 is the most effective inhibitor, followed by Arg-2, while Mem-T is ineffective (Figures 5(a) and 10(a)). Although Arg-1 and Arg-2 were designed as fibril elongation inhibitors, the two analogs together with Mem-T also have effects on the lag times for the reactions and could conceivably protect against WT-amylin cytotoxicity through a different mechanism. To unequivocally prove that Arg-1 and Arg-2 act as fibril elongation inhibitors and that Mem-T can interfere with membrane disruption by WT-amylin will require further studies.

The current results with the charge-based inhibitors are encouraging because they potentially represent a new electrostatic-based approach to inhibit amyloid fibrillization and toxicity. Clearly, the efficacy of these first-generation inhibitors could be improved. Possible strategies include substituting charged residues for the segments that form the earliest secondary structure during misfolding [36], using structural models of amylin fibrils to substitute charged residues for residues that face the surface or core of the fibril, substituting charged residues in both strands that form the amylin fibril *β*-hairpin structure [13, 37], and combining charge-based substitutions with other approaches such as the introduction of H-bond blockers [38, 39]. However, to achieve the goal of rational design, these structural approaches will require more mechanistic studies that validate the charge-based inhibitors work as envisioned in their conception. These studies in turn should aid in the development of more effective inhibitors.

## Abbreviations

DMSO: Dimethyl sulfoxide
DMEM: Dulbecco's Modified Eagle Medium
FBS: Fetal bovine serum
NMR: Nuclear magnetic resonance
SDS: Sodium dodecyl sulfate
SEM: Standard error of the mean
ssNMR: Solid-state NMR
TEM: Transmission electron microscopy
ThT: Thioflavin T
WT: Wild type.
